# Monitoring urban beaches with qPCR vs. culture measures of fecal indicator bacteria: Implications for public notification

**DOI:** 10.1186/s12940-017-0256-y

**Published:** 2017-05-12

**Authors:** Samuel Dorevitch, Abhilasha Shrestha, Stephanie DeFlorio-Barker, Cathy Breitenbach, Ira Heimler

**Affiliations:** 1 Division of Environmental and Occupational Health Sciences, University of Illinois at Chicago School of Public Health, 2121 W. Taylor St., M/C 922, Chicago, IL 60091 USA; 2 Chicago Park District, 541 N. Fairbanks Ct, Chicago, IL 60611 USA

**Keywords:** Surface water monitoring, Beach management, Fecal indicator bacteria, Quantitative polymerase chain reaction (qPCR), Water pollution

## Abstract

**Background:**

The United States Environmental Protection Agency has established methods for testing beach water using the rapid quantitative polymerase chain reaction (qPCR) method, as well as “beach action values” so that the results of such testing can be used to make same-day beach management decisions. Despite its numerous advantages over culture-based monitoring approaches, qPCR monitoring has yet to become widely used in the US or elsewhere. Considering qPCR results obtained on a given day as the best available measure of that day’s water quality, we evaluated the frequency of correct vs. incorrect beach management decisions that are driven by culture testing.

**Methods:**

Beaches in Chicago, USA, were monitored using *E. coli* culture and enterococci qPCR methods over 894 beach-days in the summers of 2015 and 2016. Agreement in beach management using the two methods, after taking into account agreement due to chance, was summarized using Cohen’s kappa statistic.

**Results:**

No meaningful agreement (beyond that expected by chance) was observed between beach management actions driven by the two pieces of information available to beach managers on a given day: enterococci qPCR results ofsamples collected that morning and *E. coli* culture results of samples collected the previous day. The *E. coli* culture beach action value was exceeded 3.4 times more frequently than the enterococci qPCR beach action value (22.6 vs. 6.6% of beach-days).

**Conclusions:**

The largest evaluation of qPCR-based beach monitoring to date provides little scientific rationale for continued *E. coli* culture testing of beach water in our setting. The observation that the *E. coli* culture beach action value was exceeded three times as frequently as the enterococci qPCR beach action value suggests that, although the beach action values for bacteria using different measurement methods are thought to provide comparable information about health risk, this does not appear to be the case in all settings.

## Background

National estimates suggest that 41.5% of the US population over the age of 16 years swims at beaches at least once per year [1]. At several marine and Great Lakes beaches, the incidence of gastrointestinal illness following swimming has been shown to increase with increasing concentrations of fecal indicator bacteria (FIB) in beach water [2, 3]. Given that tens of millions of people swim annually, the health of the public can be protected by monitoring recreational waters for FIB, and promptly issuing alerts about elevated health risks. The US Environmental Protection Agency (USEPA) has established beach monitoring and notification programs and water quality criteria, which have advanced over time as new data and methods have become available [4, 5].

The monitoring of FIB concentrations in beach water using the quantitative polymerase reaction (qPCR) has several potential significant advantages over FIB monitoring using culture methods. First, the analyses can be conducted within several hours [6], as opposed to the minimum of 18 h required by culture methods. As a result, the public can be notified promptly about elevated FIB concentrations, as opposed to the next day, when culture results are reported. Second, the National Environmental and Epidemiological Assessment of Recreational water (NEEAR), a multi-year, multi-site cohort study found that compared to the culture measures of FIB, qPCR measures of enterococci are better independent predictors of the occurrence of gastrointestinal illness among swimmers at freshwater [2] and marine [3] beaches located within several kilometers of wastewater treatment facility outfalls. Third, qPCR methods, but not culture methods, readily lend themselves to microbial source identification [7]. The general enterococci qPCR target that was measured using the NEEAR study protocol was not specific to particular fecal sources. However, source-specific targets could certainly be analyzed in parallel with (or as follow-up to) the general enterococci qPCR target. Such information could be useful in efforts to control fecal pollution sources [8] or to model risks to public health [9].

Based primarily on NEEAR, the USEPA issued Recreational Water Quality Criteria (RWQC) in 2012, which for the first time included beach action values (BAVs) for enterococci measured using the qPCR method (as well as BAVs for *E. coli* and enterococci measured using culture methods) [4]. The *E. coli* BAV of 235 colony forming units (CFU)/100 mL of water and enterococci qPCR BAV of 1000 calibrator cell equivalents (CCE)/100 mL of water are both expected to limit the gastrointestinal illness attributable to swimming to 36 cases per 1000 swimmers. If a beach manager employed both qPCR and culture testing on a daily basis, on a given day, he/she would know whether that morning’s qPCR results and the previous day’s culture results exceeded their respective BAVs. Thus, although both BAVs are meant to be equivalent, that would only be true if there had not be a day-long lag in obtaining the culture results during which water quality could change substantially.

Over the past dozen years, investigations into qPCR measures of FIB in beach water have moved progressed substantially (Table 1). Early studies involved the splitting of water samples, and testing for FIB immediately by culture, and then archiving filters for subsequent qPCR analyses.

**Table 1:** Studies of qPCR and culture measures of FIB, sorted by timing of the qPCR analyses and whether the data analyses considered policy-relevant threshold values and timeframes

	qPCR analyses		
Measures of association between qPCR and FIB culture results	Archived samples analyzed	Analyzed in real-time, results not used for public notification	Analyzed in real-time, results, used for same-day notification
Day0 qPCR and Day0 culture measures of FIB as continuous variables (correlation, linear regression analysis)	[11, 13, 14, 26–30]	[17, 31, 32]	[6, 15, 16]
Day0 qPCR and Day0 culture measures of FIB as dichotomous measures (such as BAV exceedance), agreement due to chance not addressed	[11, 13, 14, 29, 30]^a^	[17, 31, 32]^a^	[15, 16]^a^
Day1 qPCR and Day0 culture measures of FIB as dichotomous categories (such as BAV exceedance), agreement due to chance not addressed			
Day1 qPCR and Day0 culture measures of FIB as dichotomous categories (such as BAV exceedance), agreement due to chance addressed			Present study of Chicago beaches

^*a*^ *A threshold for qPCR different than the current BAV of 1000 CCE/10 mL appears to have been used*

Data from the two methods of quantifying FIB were analyzed using correlation and/or linear regression methods. Later studies evaluated concordance of threshold exceedance by the two methods. It is known that FIB concentrations change significantly on time scales of hours [10–12], making the Day0 culture results of limited value in predicting FIB concentrations on Day1. We know that the exceedance of the Day0 culture BAV is associated with exceedance of the Day0 qPCR BAV at numerous locations studied [11, 13, 14]. From the standpoint of deciding whether qPCR or culture methods should be used for public notification purposes, it is the agreement between Day0 culture BAV and Day1 qPCR BAV (not Day0 vs. Day0) this matters. Furthermore, analysis of dichotomous outcomes (exceedance of the BAV as determined by two methods) must take into account agreement due to chance. Consider a beach management program based on the outcome of rolling a die. If the die lands on the number six, an advisory is issued; for all other outcomes, no advisory is issued. If a second die were to be rolled (as a new method of testing), by chance alone the “results of testing” by the two “methods” would often agree with one another. Both methods would call for no advisory: 5/6 × 5/6, or 69.4%, of the time. Likewise, both methods would call for an advisory 1/6 × 1/6, or 2.7%, of the time. Overall, chance alone would account for agreement in 69.4% + 2.7% = 71.9% of the “days of beach testing.” Therefore, any analysis of agreement must take chance into account. Strong agreement between management decisions based on Day0 culture results and Day1 qPCR results would suggest that continued use of the low-tech (but slower) culture methods may be appropriate. Weak agreement – particularly if the need to issue an advisory to the public is not triggered by Day0 culture results when Day1 qPCR results do trigger an advisory – would indicate that the health of beachgoers would be better protected if the qPCR method were to be used.

We are aware of only three peer-reviewed publications that describe the implementation of a qPCR-based same-day beach notification program. One involved qPCR testing of 44 water samples (once per week for 11 weeks) at four New Jersey beaches [15]. The other two described a daily beach monitoring and notification program at nine southern California beaches, involving 239 samples [6, 16]. In these settings water samples were analyzed using culture and qPCR methods and public notification took place by mid-day, and the results of culture and qPCR analyses of the same water samples generally arrived at similar beach management decisions. A third study that involved 259 samples collected from 8 beaches in Door County, Wisconsin included real-time beach monitoring by qPCR [17], though the results were not used for beach notification purposes (personal communication, G. Kleinheinz). We are aware of no published studies that have evaluated the agreement between BAV exceedance by qPCR and culture measures of FIB after taking into account 1) the impacts of the 24-h delay in obtaining culture results, or 2) the agreement between results agreement due to chance.

The Chicago Park District and the University of Illinois at Chicago School of Public Health water quality laboratory have partnered on a multi-beach, multi-year beach water quality monitoring program that utilized the qPCR method, while maintaining standard culture testing. Using data collected during two beach seasons over a total of 898 beach-days (e.g., in 2015 we tested 5 beaches/day × 54 days =270 beach-days), we evaluated the agreement between the beach management decisions that result from culture and qPCR testing, taking into account the frequency of agreement expected by chance alone. The primary goals of this research were to: 1) characterize differences in public notifications about BAV exceedances between Day1 qPCR results and Day0 culture results; and, 2) identify the frequency of discrepancies in BAV exceedances using the two methods, which are meant to represent comparable degrees of health risk under the 2012 USEPA RWQC.

## Methods

### Setting

**Fig. 1:**
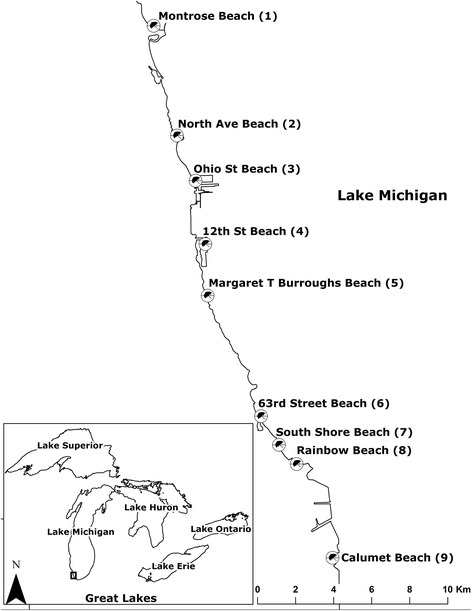
Map of beaches studied

Chicago is located on the southwestern edge of Lake Michigan (Fig. 1). The Chicago Park District maintains one of the most comprehensive beach monitoring programs in the United States at 27 public beaches, which host an estimated 20 million beach visits annually. The beaches account for much of the 42 km of Lake Michigan shoreline within the City of Chicago, and have been monitored at least 5 days per week using *E. coli* culture testing. Wastewater and stormwater from Chicago flow into the highly engineered Chicago River system, away from Lake Michigan and towards the Mississippi River system (except following infrequent extreme precipitation events). Spatial and temporal aspects of *E. coli* concentrations at these beaches have been well-characterized by researchers at the US Geological Survey Great Lakes Science Center [18–21].

### Water sampling

Nine beaches were studied, numbered from 1 through 9 (north to south, in Fig. 1). In 2015, beaches 1, 6, 7, 8 and 9 were sampled; in 2016, monitoring was extended to include beaches 1–9. In 2015, water was sampled on Tuesday-Friday from May 26 through August 28, for a total of 54 days, or 270 beach-days. In 2016 water was to be sampled Wednesday-Sunday from June 1 through September 4 at nine beaches on 70 days, or 630 beach-days. Water samples were collected in 1 L polypropylene copolymer bottles at each of two transects, approximately 100 yards apart, at each beach. At the same times and locations, water samples were collected for *E. coli* culture analyses conducted by a commercial laboratory using the defined substrate (Colilert®) method.

### Sample receipt and initial processing

Water samples were delivered to the University of Illinois at Chicago School of Public Health Water Microbiology Research Laboratory (UIC) at approximately 8:30 AM. After a chain of custody document was signed, the samples (10 per day in 2015 [five beaches x two transects/beach] and 18 per day in 2016 [nine beaches x two transects/beach]) were split into 100 mL aliquots for qPCR analysis of enterococci. The qPCR laboratory consists of a “dirty room” where filtration and measurement of physicochemical parameters take place; an “intermediate room” where DNA extraction takes place, sample DNA is loaded into plates, and where thermocyclers are located; and a “clean room” for reagent preparation.

### qPCR analysis

Laboratory analyses followed USEPA’s Method 1609 [22] and 1609.1 [23] in 2015 and 2016, respectively, with one modification: the salmon DNA sample processing control was used, but the plasmid internal amplification control was not. Briefly, the process included filtration of 100 mL through 47 mm polycarbonate filters with a 0.4 μm pore size. Following filtration, genomic DNA was extracted from the filters by bead-beating using pre-filled glass bead tubes (GeneRite, North Brunswick, NJ). In 2015 final crude genomic DNA extracts were diluted 5-fold in AE buffer and 5 μl were added, in duplicate, to 20 μl of ABI TaqMan® Environmental Master Mix 2.0 (Applied Biosystems, Foster City, CA), which contained the environmental master mix, primers, probes, bovine serum albumin, and nuclease-free water. Because of the low rate of inhibition observed in 2015 (described in detail in ‘Results’), in 2016 dilution of DNA extracts was not performed. Primers and probes (Integrated DNA Technologies, Inc., Coralville, Iowa) were as specified in Method 1609. All reactions were performed on the ABI StepOne Plus® platform.

### qPCR data quality

The qPCR data quality monitoring was based on that described in EPA Methods 1609 and 1609.1. Calibrator samples consisted of 10 μL aliquots of quantified cell concentration of laboratory-prepared, cultured *Enterococcus faecalis* (ATCC® 29,212™) stock suspensions. In 2015, 10 μL aliquots of calibrators cells were spotted onto filters directly (per Method 1609); in 2016 calibrator cells were filtered onto the membranes (as per Method 1609.1) [23]. Genomic DNA was extracted from these aliquots in the same manner as the sample DNA extracts. No-template control (NTC), method blank (MB), and sample processing control (SPC) samples were run daily on the same 96-well plate as the set of samples. All NTC, MB, SPC, and water sample reactions were performed in duplicate. Standard curves were generated from serially diluted, purified, RNA-free and spectrophotometrically quantified *E. faecalis* genomic DNA, using primers and probes to amplify and detect a sequence of DNA that codes for the *Enterococcus* large ribosomal subunit. Each dilution was analyzed in triplicate. Standard curves were run alongside the samples on a biweekly basis and a composite standard curve was generated and analyzed using linear regression [24]. *Accuracy* of qPCR data was defined by the R^2^ of standard curves, using enterococci DNA concentrations of 10, 40, 400, 4000, and 40,000 target sequence copies per reaction. *Precision* of qPCR results was evaluated by, 1) the coefficient of variation of calibrators, and 2) the coefficient of variation of sample processing controls in method blanks. *Inhibition* of the qPCR reaction may be caused by substances found in the water environment, and can be identified by an increase in the number of amplification cycles needed before the salmon DNA SPC is detected (in other, words, the cycle threshold [Ct] is increased). EPA Method 1609 notes that a difference in detecting the SPC in calibrator and water samples of greater than 3 cycles of amplification (or ΔCt_SPC_ ≥ 3) “…suggests that total DNA recovery was < ~10% and therefore potentially unacceptably high levels of enterococci may not be detectable due to the very low recovery.” We summarized the frequency of samples with ΔCt_SPC_ ≥ 3 and of 2 ≤ ΔCt_SPC_ < 3 cycles.

*E. coli* culture testing was performed by a commercial laboratory using the Colilert® method (IDEXX Laboratories, Westbrook, ME) and results are reported as a ‘most probable number’ (MPN) per 100 mL of water. The upper limit of quantification of this method is 2420 MPN/100 mL. By convention, the Chicago Park District has used the geometric mean (rather than the arithmetic mean) of concentrations of bacteria in water samples collected from the two transects at each beach to arrive at a single measure of bacteria concentrations. In this report, we follow that convention.

### Data analysis

The normality of distributions of original scale and log10-transformed water quality data were evaluated using the Kolmogorov-Smirnov test, using *p* > 0.15 to indicate normality. Pearson correlation coefficients of normally-distributed data were described. We sought to characterize the relationship between exceedance of the *E. coli* culture BAV of 235 MPN/100 mL and exceedance of the enterococci qPCR BAV of 1000 calibrator cell equivalents (CCE)/100 mL. Agreement between exceedance of the two BAVs (*E. coli* culture, enterococci qPCR) were characterized by Cohen’s Kappa, which takes into account the expected agreement between assessments due to chance alone. The following terminology was used to describe agreement based on Kappa: 0–0.20, none; 0.21–0.39, minimal; 0.40–0.59, weak; 0.60–0.79, moderate; 0.80–0.89, strong; ≥0.90, near perfect [25]. BAV exceedance agreement analyses were conducted for Day0 culture and Day1 qPCR results, as well as for Day0 culture and Day0 qPCR results. Because Day1qPCR results reflect Day1 water quality (as opposed to prior day water quality) and because qPCR measures of enterococci were stronger independent predictors of health outcome than culture measures in NEEAR, we considered Day1 enterococci qPCR results to be the gold standard against which we evaluated the frequency of correct vs. incorrect beach management decisions. Data analyses were performed using SAS version 9.4 (SAS Institute, Cary, NC).

## Results

A total of 1796 water samples (540 in 2015 and 1256 in 2016) were collected and analyzed, representing 99.8% of all planned samples (water samples could not be collected on a single beach-day due to hazardous weather conditions, and on another date, a laboratory error resulted in no usable data from both transects of a single beach). As summarized in Table 2, data quality demonstrated both accuracy and precision. In 2015 two samples (0.37% of all transect-specific samples) were inhibited (ΔCt_SPC_ ≥ 3). The two samples were collected from different transects (on different dates), allowing the use of data from the non-inhibited transect sample for beach management decisions. Inhibition was somewhat more frequent in 2016, observed in 14 (1.1%) of transect-specific samples. Of the 14 transect-specific inhibited samples, four involved both transects of two beaches. As a result, qPCR results were not available on two beach-dates due to inhibition. The other 10 inhibited samples impacted only a single transect per beach, and on those dates, the results of the non-inhibited sample from the other transect were used for beach management purposes. Thus, of the planned use of qPCR data for beach management at 270 + 630 = 900 beach-days (in 2015 and 2016, respectively), data were used for beach management at 270 + 624 = 894 (or 99.3% of the planned) beach-days.

**Table 2:** qPCR data quality summary

Data quality metric	2015		2016	
Accuracy in composite standard curves				
Number of standard curves	9		9	
R^2^	0.996		0.999	
Amplification efficiency	1.933		2.097	
Precision: calibrator samples CV				
Number of samples	55		71	
Enterococci target CV	2.05%		1.33%	
SPC target CV	1.11%		1.41%	
Inhibition	Frequency	Percent	Frequency	Percent
ΔCt_SPC_ < 2	536	99.26	1210	96.3%
2 ≤ ΔCt_SPC_ < 3	2	0.37%	32	2.5%
ΔCt_SPC_ ≥ 3	2	0.37%	14	1.1%
Total	540	100%	1258	100%

*CV* coefficient of variation

### Water quality

Enterococci qPCR CCE/100 mL values were log-normally distributed (Kolmogorov-Smirnov *p* > 0.15) and *E. coli* MPN/100 mL values approximated a lognormal distribution (Kolmogorov-Smirnov *p* = 0.09). For that reason, median and the central 90% range (5th and 95th percentile values) are summarized in Table 3 (rather than mean and standard deviation). Neither the median CCE nor median MPN values approached their respective BAVs. Exceedance of the *E. coli* culture BAV was approximately three times as common as exceedance of the qPCR BAV (8.5% for qPCR vs. 24.8% for *E. coli* culture in 2015; 5.0% for qPCR vs. 21.1% for *E. coli* culture in 2016). Statistically significant correlations were observed between the 686 pairs of log10 transformed CCE/100 and MPN/100 values (Pearson rho = 0.65; *p* < 0.001). Fewer *E. coli* culture data points (*n* = 687) were available than qPCR results (*n* = 894), as *E. coli* testing was only performed by the commercial laboratory on weekends if the prior day’s result for that beach exceeded the BAV.

**Table 3:** Water quality and exceedance of beach action values (BAVs) at beaches, 2015 and 2016

	qPCR testing (enterococci)						Culture testing (*E. coli*)					
	2015			2016			2015			2016		
Beach	N	Median (5th, 95th %)	% Excd.	N	Median (5th, 95th %)	% Excd.	N	Median (5th, 95th %)	% Excd.	N	Median (5th, 95th %)	% Excd.
1	54	144.3 (29.6, 2210.6)	9.3	70	116.9 (8.2, 1724.1)	10.0	54	172 (29, 1540)	38.9	52	138 (20, 1540)	34.6
2				67	39.8 (5.2, 225.0)	0				41	37 (8, 177)	2.4
3				70	43.9 (4.9, 1109.4)	5.7				44	35 (3, 318)	11.4
4				70	46.8 (4.4, 562.9)	1.4				43	45 (6, 303)	11.6
5				68	79.4 (5.1, 1087.6)	5.9				46	90 (6, 1311)	23.9
6	54	85.1 (19.5, 1201.0)	9.3	70	120.2 (10.4, 1399.7)	12.9	54	40 (5, 738)	16.7	49	139 (18, 848)	32.7
7	54	157.6 (21.8, 1006.8)	5.6	69	76.5 (11.9, 570.6)	1.4	54	79 (12, 398)	14.8	42	48 (14, 387)	9.5
8	54	162.3 (27.1, 1383.8)	9.3	70	94.6 (7.1, 918.4)	4.3	54	104 (8, 729)	22.2	50	91 (5, 1646)	26.0
9	54	239.9 (29.3, 1468.3)	9.3	70	77.9 (5.9, 739.6)	2.9	54	66 (8, 1304)	31.5	50	111 (1, 1939)	30.0
ALL	270	154.5 (26.6, 1424.6)	8.5	624	74.1 (7.0, 918.4)	5.0	270	88 (8, 1,21)	24.8	417	74 (7, 1137)	21.1

‘% Excd’ refers the percent exceedance of the respective beach action value

### Public health action

Water quality information available to beach managers on a given day by 1:00 PM – the Day1 qPCR results and Day0 culture results – generally led to similar beach management actions (Table 4). The Day0 culture results generally did not trigger a beach advisory, and this generally corresponded with the Day1 qPCR results, which also did not trigger a beach advisory. Considering Day1 qPCR results as the gold standard, the Day0 culture generated correct results 71.3% of the time. However, had the Day1 qPCR result not been available, Day0 culture results would have triggered unnecessary beach advisories 24% of the time (“false alarms”). Critically, on 4.7% of the beach-days, Day0 culture results would have resulted in a “failure to act” (meaning, failure to trigger advisories) when advisories were needed (based on exceedance of the Day1 qPCR BAV). At one beach, ‘failure to act” would have occurred 6.8% of the time. Overall, Day0 culture results were more likely to result in a “failure to act” (*n* = 32) than a “correct advisory” (*n* = 13).

**Table 4:** Beach management accuracy resulting from use of *E. coli* culture results available to beach managers (collected on Day0), using Day1 qPCR BAV *exceedance as the gold standard*

Beach	Correct: advisory	Correct: no advisory	Failure to act	False alarm	Total
1	4 (3.8)	58 (55.2)	6 (5.7)	37 (35.2)	105 (100)
2	0 (0.0)	37 (92.5)	0 (0.0)	3 (7.5)	40 (100)
3	1 (2.3)	36 (81.8)	3 (6.8)	4 (9.1)	44 (100)
4	0 (0.0)	36 (83.7)	0 (0.0)	7 (16.3)	43 (100)
5	0 (0.0)	33 (71.7)	2 (4.4)	11 (23.9)	46 (100)
6	5 (5.0)	67 (67.0)	5 (5.0)	23 (23.0)	100 (100)
7	0 (0.0)	77 (81.9)	4 (4.3)	13 (13.8)	94 (100)
8	2 (2.0)	66 (64.7)	6 (5.9)	28 (27.5)	102 (100)
9	1 (1.0)	59 (57.8)	6 (5.9)	36 (35.3)	102 (100)
All	13 (1.9)	469 (69.4)	32 (4.7)	162 (24.0)	676 (100)

While the 71.3% concordance of Day1 qPCR and Day0 culture beach actions may seem high, it can be explained entirely by chance, with Cohen’s kappa =0.01 (Table 5). There was “minimal” agreement (kappa = 0.22 [95% confidence interval 0.14, 0.30] between the need for beach action based on results of Day0 qPCR and Day0 culture testing performed on water samples collected at the same time (even though the culture results would become available the day after the qPCR results are available). The degree of agreement varied substantially among beaches, from none or borderline at seven of the nine beaches, to Kappa = 0.44 (0.23, 0.64) or “weak” at beach #6.

**Table 5:** Agreement between beach management that result from *E. coli* culture testing and, as the gold standard, enterococci qPCR testing

		Prior day sample	Same day sample	
Beach	*n*	Description, Kappa (95% LCL, UCL)	*n*	Description, Kappa (95% LCL, UCL)
1	105	None, 0.00 (−0.13, 0.14)	106	Minimal, 0.26 (0.10, 0.41)
2	40	undefined	40	undefined
3	44	None, 0.13 (−0.25, 0.52)	44	None, 0.13 (−0.25, 0.52)
4	43	undefined	43	undefined
5	46	None, −0.08 (−0.18, 0.02)	46	None, 0.25 (−0.04, 0.54)
6	100	None, 0.14 (−0.05, 0.32)	103	Weak, 0.44 (0.23, 0.64)
7	94	Inverse, −0.07 (−0.12, −0.02)	96	None, 0.07 (−0.16, 0.29)
8	102	None, −0.02 (−0.16, 0.12)	104	None, 0.14 (−0.05, 0.33)
9	102	None, −0.08 (−0.18, 0.03)	104	None, 0.11 (−0.05, 0.26)
ALL	676	None, 0.01 (−0.04, 0.07	686	Minimal, 0.22 (0.14, 0.30)

Left half of the table: agreement between Day1 qPCR results and Day0 *E. coli * culture results. Right half of the table: agreement between Day0 *E. coli * culture results and Day0 qPCR results. “Undefined”: no qPCR exceedance

## Discussion

In the largest study to date of the application of qPCR methods for monitoring and public notification of beach water quality, precise and accurate data were generated within 4.5 h of sample receipt. The two measures of FIB available to beach managers by1:00 PM on a given day to evaluate BAV exceedance (the Day1 qPCR results and Day0 *E. coli* culture results) agreed with one other no more than would be expected by chance. Despite the fact that the qPCR BAV and the *E. coli* culture BAV are meant to provide similar degrees of public health protection, water samples collected at the same times and places (Day0 culture and. Day0 qPCR) that were analyzed by both methods exceeded the culture BAV more than three times as frequently as they did the qPCR BAV (22.6% vs. 6.6%).

The past 12 years have seen great strides toward the application of qPCR methods to beach monitoring and notification purposes. Studies of qPCR methods for water testing have advanced from laboratory analysis of frozen filters [2, 11, 14, 26–28] and data analyses using correlations or linear regression methods. Some of those studies [11, 14, 29–32] as well as newer investigations [13, 16, 17] evaluated associations between the exceedance of threshold values for culture and qPCR methods. However, because these studies generally did not include a time series of daily measurements, comparisons were limited to Day0 culture threshold exceedance and Day0 qPCR threshold exceedance. Our finding that, after accounting for chance, Day0 culture and Day1 qPCR results had no meaningful agreement regarding BAV exceedance (Table 5), leaves beach managers in settings like Chicago with little scientific basis for the continued use of culture testing for daily public notification purposes. The fact that, by contrast, Day1 culture BAV exceedances (but not Day0 culture BAV exceedances) showed some agreement with Day1 qPCR exceedances is consistent with findings from other studies that FIB concentrations change significantly on time scales of hours [10–12]. In our setting, inhibition of qPCR in samples from both beach transects was rare. If inhibition were common and the inhibited results showed low CCE values, the availability of a ‘back-up’ source of information, such as hydrologic model output (but not Day0 culture results) may be useful.

While qPCR monitoring has clear advantages, the challenges to implementing beach monitoring and notification using qPCR methods described by Griffith and Weisberg [6] persist. These include the proficiency of laboratory analysts to perform the more technically challenging testing (relative to the simplicity of defined substrate culture methods), concerns about inhibition of qPCR reactions, the need to establish trust with stakeholders who are not familiar with qPCR testing but are familiar with culture testing, and the costs of establishing and operating qPCR laboratory [6]. We did not collect data that would support an analysis of total costs and total benefits of using a central laboratory to monitor water quality at multiple beaches through a partnership between a local government entity and a university. However, any such analysis should include the public health burden of illness associated with erroneous decisions that result from the use of prior-day culture results.

The USEPA 2012 Recreational Water Quality Criteria [4] and the 2014 National Beach Guidance Document and Required Performance Criteria [33] consider the *E. coli* culture BAV (of 235 CFU/100 mL), the enterococci culture BAV (of 70 CFU/100 mL), and the enterococci qPCR BAV (of 1000 CCE/100 mL) as providing a comparable degree of health protection (36 illness of gastrointestinal illness attributable to water recreation per 1000 swimmer or waders). Our work demonstrates that at Chicago beaches, the BAVs are not in alignment with one another, as the exceedance of the *E. coli* culture BAV occurred three times more frequently than exceedance of the enterococci qPCR BAV. Several prior studies have found varying degrees of concordance between the exceedance of the respective BAVs by the two methods. A recent multi-site study in which 367 river samples were analyzed by *E. coli* culture (immediately) and enterococci qPCR (after freezing and storage) found comparable frequencies of BAV exceedance between the Day0 results of generated by the two methods [13]. At some of the same beaches that we studied (but several years earlier), qPCR BAV exceedance was less common than the *E. coli* culture BAV exceedance, consistent with our findings [30]. By contrast, in three beaches on an inland lake in Ohio, USA, the *E. coli* BAV was exceeded 24% of 59 samples, while the enterococci qPCR BAV was exceeded in 100% of 49 samples [29]. At beaches in Door County, Wisconsin, the overall rate of *E. coli* culture BAV exceedance was 10%, compared to 24% rate of enterococci qPCR BAV exceedance, though these rates varied substantially across the seven beaches studied [17]. Policy makers may need to consider this non-equivalence in developing guidance for beach managers who may be confronted with the possibility of exceeding a BAV (or water quality standard) based on one method of measurement (in our case, *E. coli* culture), but not another measurement method (enterococci qPCR).

Strengths of this study include: 1) the relatively large number of beach-dates of testing, 2) the real-time application of qPCR testing, 3) the comparison of public health decision-making driven by the two methods, 4) the analyses of the two pieces of information available to beach managers on Day1 (the Day1 qPCR and Day0 culture results) rather than the results of analyses of the same water samples by the two methods (both Day0), and, 5) the accounting for chance in analyses of agreement between the public health action that results from the use of the two methods.

Our findings are subject to several limitations. First, as noted, the engineered Chicago River system protects Lake Michigan and its beaches from point sources of fecal pollution, making the setting of the present study relatively unique. Thus, the generalizability of our main findings - the lack of agreement between Day1 beach management actions based on Day1 qPCR results and Day0 culture results, and the discrepancies between BAV exceedance of water from the same beaches as the same times tested using both methods - to other settings is not known. It is possible that at beaches impacted significantly by wastewater discharges, agreement between methods may be better, the impact of the 24-h lag between culture and qPCR results may be less significant, and samples tested by both methods may generate greater concordance between BAV exceedance results of culture and qPCR methods. We compared the results of *E. coli* culture to the results of qPCR measures of enterococci. *E. coli* testing, rather than enterococci testing, is widely used at Great Lakes beaches [34]. The health risks of swimming in water in which FIB measures are at the BAV are meant to be comparable regardless of whether the BAV is enterococci by culture, enterococci by qPCR, or – at Great Lakes beaches – *E. coli* by culture. However, it is not known to what degree our results would have been different had enterococci, rather than *E. coli*, been analyzed by culture. While we referred to the Day0 qPCR results as the gold standard or reference for data analysis purposes (as they reflect same day water quality), we do not wish to suggest that FIB monitoring by culture is “sub-standard” or that it should be discontinued by jurisdictions that lack the resources to implement (or share in the implementation of) qPCR monitoring. We used the geometric mean of qPCR results from the two transects per beach, given the lognormal distribution of CCE values. Had we used the arithmetic mean of the results from the two transects to determine whether the BAV had been exceeded, there would have been 66 exceedances of the course of the two years rather than the 54 that occurred using the geometric mean, a 22% increase in frequency. Thus, jurisdictions that wish to be more conservative, in addition to using a lower threshold than 1000 CCE/100 mL to notify the public, could use the more conservative arithmetic mean of paired samples.

## Conclusion

Monitoring multiple beaches using qPCR methods can generate precise and accurate data for timely public notifications regarding beach water quality. Results of prior-day *E. coli* culture testing were no better than chance in predicting the exceedance of the qPCR BAV. “Becoming uncultured” may be appropriate in Chicago, as it would prevent failures to notify the public about beach action value exceedances. *E. coli* culture testing of beaches (on the same day) led to three times the number of BAV exceedance as did enterococci qPCR testing of beach water. It is not known whether similar results would have been obtained at marine beaches or those significantly impacted by wastewater.

## Abbreviations

BAV: Beach action value
CCE: Calibrator cell equivalent
CFU: Colony forming units
CV: Coefficient of variation
FIB: Fecal indicator bacteria
MB: Method blank
MPN: Most probable number
NEEAR: National Environmental and Epidemiological Assessment of Recreational water
NTC: No template control
qPCR: Quantitative polymerase chain reaction
RWQC: Recreational Water Quality Criteria
USEPA: United States Environmental Protection Agency
